# Identification of a Ferroptosis-Related LncRNA Signature as a Novel Prognosis Model for Lung Adenocarcinoma

**DOI:** 10.3389/fonc.2021.675545

**Published:** 2021-06-23

**Authors:** Lu Lu, Le-Ping Liu, Qiang-Qiang Zhao, Rong Gui, Qin-Yu Zhao

**Affiliations:** ^1^ Department of Blood Transfusion, The Third Xiangya Hospital of Central South University, Changsha, China; ^2^ College of Engineering and Computer Science, Australian National University, Canberra, ACT, Australia

**Keywords:** lung adenocarcinoma, ferroptosis, lncRNAs, prognosis, risk score

## Abstract

Lung adenocarcinoma (LUAD) is a highly heterogeneous malignancy, which makes prognosis prediction of LUAD very challenging. Ferroptosis is an iron-dependent cell death mechanism that is important in the survival of tumor cells. Long non-coding RNAs (lncRNAs) are considered to be key regulators of LUAD development and are involved in ferroptosis of tumor cells, and ferroptosis-related lncRNAs have gradually emerged as new targets for LUAD treatment and prognosis. It is essential to determine the prognostic value of ferroptosis-related lncRNAs in LUAD. In this study, we obtained RNA sequencing (RNA-seq) data and corresponding clinical information of LUAD patients from The Cancer Genome Atlas (TCGA) and Gene Expression Omnibus (GEO) database and ferroptosis-related lncRNAs by co-expression analysis. The best predictors associated with LUAD prognosis, including C5orf64, LINC01800, LINC00968, LINC01352, PGM5-AS1, LINC02097, DEPDC1-AS1, WWC2-AS2, SATB2-AS1, LINC00628, LINC01537, LMO7DN, were identified by Least Absolute Shrinkage and Selection Operator (LASSO) Cox regression analysis, and the LUAD risk prediction model was successfully constructed. Kaplan-Meier analysis, receiver operating characteristic (ROC) time curve analysis and univariate and multivariate Cox regression analysis and further demonstrated that the model has excellent robustness and predictive ability. Further, based on the risk prediction model, functional enrichment analysis revealed that 12 prognostic indicators involved a variety of cellular functions and signaling pathways, and the immune status was different in the high-risk and low-risk groups. In conclusion, a risk model of 12 ferroptosis related lncRNAs has important prognostic value for LUAD and may be ferroptosis-related therapeutic targets in the clinic.

## Introduction

Lung cancer is the malignancy with the highest mortality rate worldwide, with approximately 25% of cancer patients dying from lung cancer (1). Non-small-cell lung cancer (NSCLC) is the most common type, accounting for approximately 85% of all lung cancers (2). NSCLC is divided into three main subtypes: squamous cell carcinoma, adenocarcinoma and large-cell carcinoma (3). Lung adenocarcinoma (LUAD) is the most prevalent histotype of NSCLC (4). Despite the rapid development of molecular biology and precision medicine, the 5-year overall survival (OS) rate for patients with LUAD is only 15% (2). Furthermore, because lung cancer is a highly heterogeneous tumor with complex molecular mechanisms, many molecular targeted drugs are ineffective in some patients, which poses a great challenge for the treatment of lung cancer (5, 6). Therefore, it is of great importance to identify specific tumor factors and provide new biomarkers for accurate diagnosis, individualized treatment, and prognosis prediction of LUAD.

Long non-coding RNAs (lncRNAs) are RNAs with a molecular weight of more than 200 nucleotides. Although lncRNAs are not involved in protein translation, they play a key role in gene regulation. In different biological and physiopathological contexts, lncRNAs can affect gene expression by regulating chromatin function, regulating the assembly and function of membrane-free nucleosomes, as well as changing the stability and translation of cytoplasmic mRNAs and interfering with signaling pathways (7). In recent years, with the development of high-throughput sequencing, people have discovered a large number of non-coding genes play an important role in the development and progression of tumors (8). Studies have demonstrated that lncRNAs are involved in cell growth, invasion and metastasis (9, 10), as well as tumor angiogenesis (11). Many lncRNAs are specifically expressed in different tumors and are involved in complex biological processes by interacting with DNA, RNA and proteins (12). The key role of lncRNAs in tumors indicates their promise as new targets for precise cancer therapy.

Ferroptosis is a unique cell death mechanism distinguished from apoptosis. Ferroptosis is characterized by iron-dependent peroxidation and is associated with oxidative polyunsaturated fatty acids (PUFAs), reactive oxygen species (ROS) and Lipoperoxide (13). In recent years, studies have demonstrated that ferroptosis is an important regulatory mechanism for tumor growth and is important for chemoradiotherapy and immunotherapy of tumors (14). Therefore, combinations with agents targeting iron death signaling could improve the anti-tumor efficacy of these therapeutic approaches. Peng Chen et al. demonstrated that Erianin, a natural product isolated from Dendrobium Chrysotoxum Lindl, can induce ferroptosis in lung cancer cells through Ca2 +/CaM signaling pathway and exert anti-tumor effects (15). In addition, it was demonstrated that lncRNA LINC00336 is highly expressed in lung cancer, and acts as a competitive endogenous RNA to function as an oncogene. Notably, the carcinogenesis of lncRNA LINC00336 is associated with ferroptosis (16). At present, the mechanism of ferroptosis-related lncRNAs in lung cancer progression has not been clarified, and their important significance in lung cancer treatment and prognosis needs to be further elucidated.

The tumor immune microenvironment (TIME) is associated with iron metabolism and homeostasis *in vivo*, while ferroptosis plays a key role in tumor immunity (17). Ferroptosis can expose tumor antigens thereby improving the immunogenicity of TIME and the effect of immunotherapy (18). Studies have shown that inhibition of system $x_{c}^{-}$ (a cystine/glutamate antiporter) expression by interferon-γ (IFNγ) and CT8 + T cells in TIME can induce ferroptosis in cancer cells (19, 20). The effect of ferroptosis on tumors involves a variety of immune cells and immune factors. Therefore, it is important to explore biomarkers associated with tumor immunity and ferroptosis for immunotherapy of lung cancer.

Aberrant expression of lncRNAs and abnormal alterations in ferroptosis are both common phenomena in tumor cells and are associated with tumor progression (14, 21). However, their direct interconnection and role in lung cancer are unknown and require further investigation. The purpose of this study is to explore the significance and molecular mechanism of ferroptosis-related lncRNAs in LUAD for the first time by bioinformatics methods and find biomarkers with potential utilization value so as to improve the clinical efficacy and improve the prognosis of LUAD patients. In this study, we used TCGA and GEO databases to search for ferroptosis-related lncRNAs that are differentially expressed in LUAD, and identified 12 lncRNAs that are closely related to LUAD prognosis and successfully constructed a LUAD risk prediction model. In addition, the acting mechanism of ferroptosis-related lncRNAs in tumor progression was further mined by functional analysis and immune infiltration analysis to provide new insights into the prognosis and immunotherapy of LUAD.

## Materials and Methods

### Patients and Datasets

The RNA sequencing (RNA-seq) data and corresponding clinical information of patients with LUAD were downloaded from The Cancer Genome Atlas (TCGA) database and Gene Expression Omnibus (GEO) database. The data from TCGA database was used as a training cohort to establish the multi-lncRNA prognosis model, and the data from GEO (GSE30219, GES31210, GSE31546) as a validation cohort was used to test the predictive power of the risk score.

### Identification of Ferroptosis-Related LncRNAs

Ferroptosis-related genes obtained by summarizing previous literature (22–24) and were provided in **Table S1**. First, the lncRNA and mRNA expression profiles were extracted separately from TCGA database. Then, the ferroptosis-related lncRNA profiles were excerpted from the ferroptosis-related genes based on the co-expression analysis (25). The ferroptosis-related lncRNAs was obtained through correlation analysis between the expression levels of the lncRNAs and ferroptosis-related genes, using Pearson correlation coefficients (coefficients > 0.40, P < 0.05). In addition, we explored regulatory mechanisms of ferroptosis-related lncRNA. We extracted ferroptosis-related lncRNA significantly correlated with co-expression and ferroptosis genes to construct the regulatory network and used Cytoscape software to display the ferroptosis genes and ferroptosis-related lncRNA regulatory network.

### Construction of the Risk Score

First, differential expression analysis of ferroptosis-related lncRNAs was performed by using a “limma” R package, and differentially expressed lncRNAs were selected with an absolute log2-fold change (FC) > 1 and an adjusted P value < 0.01 (26). Next, overall survival was set as the clinical endpoint in our study. Then, univariate Cox regression was applied to identify ferroptosis*-*related lncRNAs related to overall survival for model construction. LncRNAs with a P value less than 0.01 were considered as significant prognostic signatures. A hazard ratio (HR) was used to determine whether lncRNAs are risk factors (HR>1) or protective factors (HR<1).

The set of differentially expressed lncRNAs and the set of prognosis-related ones were intersected, and differentially expressed lncRNAs with prognosis value were selected. Based on these genes, we used the Least Absolute Shrinkage and Selection Operator (LASSO) Cox regression analysis by using the “glmnet” R package to identify the best prognostic signature (27, 28). Specifically, the LASSO Cox regression was used to fit the overall survival of LUAD patients based on selected lncRNAs. During regression, the key lncRNAs were assigned non-zero coefficients and were selected to develop a risk score. Then, our risk score was constructed by using LASSO regression coefficients and expression levels of the key lncRNAs (29, 30). LUAD patients from the TCGA database, as a training cohort, were divided into high-risk group and low-risk group for further study based on the median score as the cutoff. Last, the patients in the GEO cohorts were also divided into two risk groups based on a cohort-specific cut-off.

### Validation of the Risk Score

Using the “survival” R package and “survivalROC” R package, the area under the time dependent receiver operating characteristic (AUROC) were assessed to evaluate the predictive power of this prognostic model (26). Besides, the survival analysis of both high-risk group and low-risk group were performed by “Kaplan–Meier” and the “survival” package log-rank test (30). Based on the expression of prognostic ferroptosis-related lncRNA, principal component analysis (PCA) was performed with the “prcomp” function of the “stats” R package (31). Besides, t-distributed stochastic neighbor embedding (t-SNE) was implemented to explore the distribution of different groups using the “Rtsne” R package (23). The same analyses were performed on the validation cohort (the data from GEO database) for validation.

### Independent Prognostic Analysis

Univariate and multivariate Cox regression analyses was performed to evaluate whether the prognostic model was independent of other traditional clinical characteristics (including age, gender, smoking, race and TNM stage) in predicting OS of patients with LUAD. The independent prognostic analysis was done on the TCGA cohort and on the GEO cohorts.

### Functional Enrichment Analysis

After identifying the lncRNA signature correlating with LUAD prognosis, the gene ontology (GO) and Kyoto encyclopedia of genes and genomes (KEGG) pathways were used to assess the biological roles of the prognostic candidates by the “clusterProfiler” R package (31). To further investigate the potential immunomodulatory mechanism of lncRNA in the regulation of tumor-infiltrating immune cells, the single-sample gene set enrichment analysis (ssGSEA) was performed to assess the infiltration abundance between the high-risk group and low-risk group of the TCGA cohort.

### Immunogenomic Landscape Analyses

Computational methods were used to evaluate the immune infiltration and functions, including ESTIMATE (32), TIMER (33), MCP-counter (34), CIBERSORTx (35), and single-sample gene set enrichment analysis (ssGSEA) in an attempt to comprehensively analyze the immune differences between the two groups of the TCGA cohort (35). The immune infiltration and functions were compared between the high- and the low-risk groups by using the two-sample Wilcoxon test.

## Results

### Differentially Expressed mRNAs and LncRNAs Associated With Prognosis

**Figure 1:**
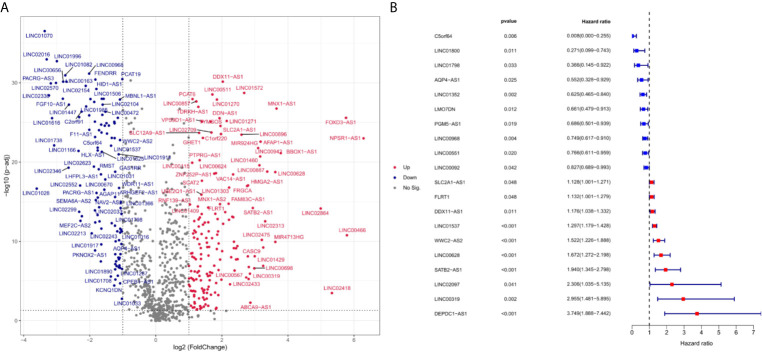
Differentially expressed ferroptosis-related lncRNA between LUAD and normal samples (log2 fold change>2, adjusted p-value < 0.01). Compared with normal samples, 49 ferroptosis-related lncRNAs were upregulated and 43 ferroptosis-related lncRNA downregulated in LUAD samples. **(A)** is a volcano map for differentially expressed ferroptosis-related lncRNAs. Red dots represent significantly upregulated expressed genes and green dots represent significantly downregulated expressed genes. **(B)** indicates the HR (95% Cl) and p-value of selected lncRNAs by univariate Cox proportional hazards. Blue dots represent protective factors, and red dots represent risk factors.

Expression data and clinical information of four cohorts were downloaded from TCGA and GEO. The baseline characteristics were summarized in **Table 1**. The first cohort consisted of 535 LUAD and 59 normal samples from TCGA, and was used for differential expression analysis. 17122 lncRNAs were screened, and 282 lncRNAs were differentially expressed between LUAD and normal groups. The expression profiles of differentially expressed lncRNAs (DElncRNAs) were visualized in the form of volcano map and heatmap (**Figures 1A** and **S1**, respectively). As shown in **Figures 1A** and **S1**, 171 lncRNAs were upregulated, and 111 lncRNAs were downregulated in LUAD samples. 119 DElncRNAs were associated with the overall survival of LUAD patients using univariate Cox regression. The lncRNAs with the 10 highest and the 10 lowest Hazard Ratios (HRs) were reported in **Figure 1B**.

**Table 1:** The baseline characteristics of the patients in TCGA and GEO cohorts.

		TCGA	GSE31210	GSE30219	GSE31546
LUAD Samples		535	226	85	17
Age, mean (SD)		65.56 (10.09)	59.58 (7.40)	61.49 (9.28)	–
Gender, n (%)	Male	249 (46.54)	105 (46.46)	66 (77.65)	3 (17.65)
Ever-smoker, n (%)	0.0	–	115 (50.88)	–	4 (23.53)
	1.0	–	111 (49.12)	–	13 (76.47)
Stage, n (%)	I	294 (55.79)	168 (74.34)	71 (83.53)	–
	II	123 (23.34)	58 (25.66)	13 (15.29)	–
	III	84 (15.94)	0 (0.00)	1 (1.18)	–
	IV	26 (4.93)	0 (0.00)	0 (0.00)	–
T, n (%)	1	175 (32.71)	–	71 (83.53)	11 (64.71)
	2	289 (54.02)	–	12 (14.12)	5 (29.41)
	3	49 (9.16)	–	2 (2.35)	0 (0.00)
	4	19 (3.55)	–	0 (0.00)	0 (0.00)
	X	3 (0.56)	–	0 (0.00)	1 (5.88)
N, n (%)	0	348 (65.17)	–	82 (96.47)	15 (88.24)
	1	95 (17.79)	–	3 (3.53)	1 (5.88)
	2	74 (13.86)	–	0 (0.00)	0 (0.00)
	3	2 (0.37)	–	0 (0.00)	0 (0.00)
	X	15 (2.81)	–	0 (0.00)	1 (5.88)
M, n (%)	0	361 (68.24)	–	85 (100.00)	–
	1	25 (4.73)	–	0 (0.00)	–
	X	143 (27.03)	–	0 (0.00)	–

### Construction of a Prognostic Model in the TCGA Cohort

**Figure 2:**
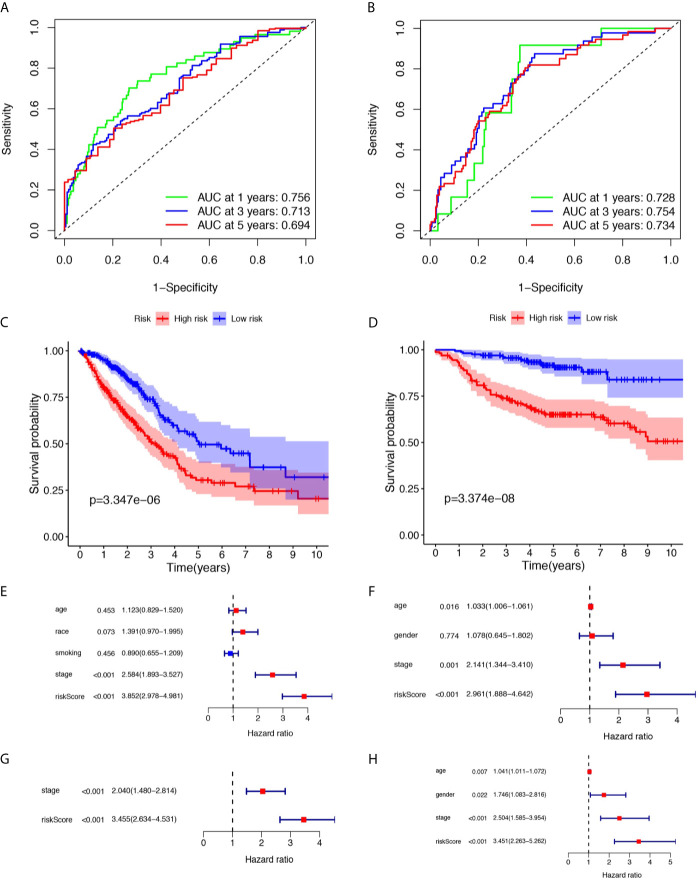
Prognostic analysis of the risk score in the TCGA and three GEO cohorts. **(A, B)** Time-dependent receiver operating characteristic curves assess the prognostic performance of the risk score in TCGA and three GEO cohorts respectively. **(C, D)** Kaplan-Meier curves display the overall survival of patients in the high- and low-risk group in TCGA and three GEO cohorts respectively. **(E, F)** The univariate Cox regression analysis of the associations between the risk scores and clinical parameters and the overall survival (OS) of patients in TCGA and three GEO cohort. **(G, H)** The multivariate Cox regression analysis of the associations between the risk scores and clinical parameters and the OS of patients in TCGA and three GEO cohorts.

**Figure 3:**
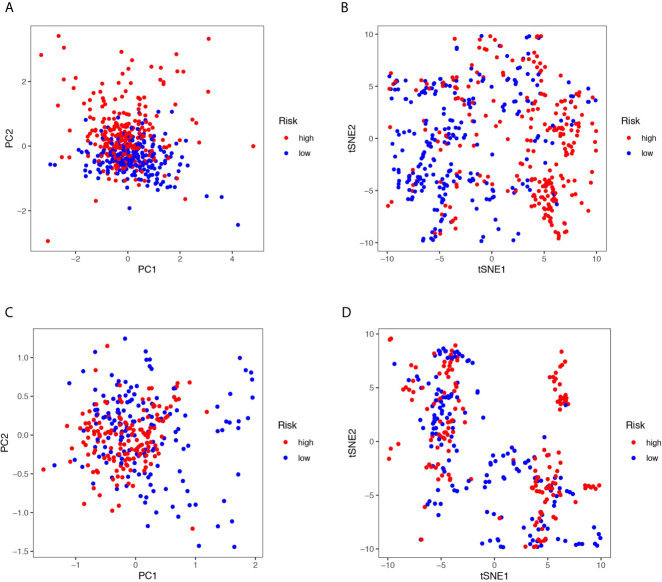
**(A, C)** Principal component analysis (PCA) plot in the TCGA and three GEO cohorts. **(B, D)** t-distributed stochastic neighbor embedding (tSNE) analysis in the TCGA and three GEO cohorts.

12 ferroptosis-related lncRNAs were identified in the LASSO Cox regression model. According to this risk model, we used a special formula (**Table S2**) to calculate the risk score of each sample. Samples were classified into high- and low-risk groups by comparing their risk scores to the median score in the TCGA cohort and GEO cohort. As shown in **Figures 2A, B**, the areas under the time-dependent ROC of the TCGA cohort are 0.756, 0.713, and 0.694 for 1-, 3-, and 5-years survival, respectively. The areas under the time-dependent ROC of the GEO cohort are 0.728, 0.754, and 0.734 for 1-, 3-, and 5-years survival. The Kaplan Meier analysis with log-rank tests revealed a significant difference in the overall survival (p<0.01) between the two groups (shown in **Figures 2C, D** **)** in two cohorts. The patients in the high-risk group had a higher probability of death earlier than patients in the low-risk group. The univariate and multivariate Cox regression analyses were performed to evaluate whether clinical parameters and the risk score are independent prognostic factors of OS (**Figures 2E–H** **)**. As shown in **Figures 2G, H** the stage and the risk score were proven to be independent factors for LUAD in TCGA cohort (HR>1, p<0.05). The age, gender, stage, and risk score were proven to be independent factors for LUAD in the GEO cohort (HR>1, p<0.05). We also indicated that LUAD patients in different risk groups were distributed in two directions by PCA and t-SNE analyses (**Figure 3**).

### Functional Enrichment Analyses

**Figure 4:**
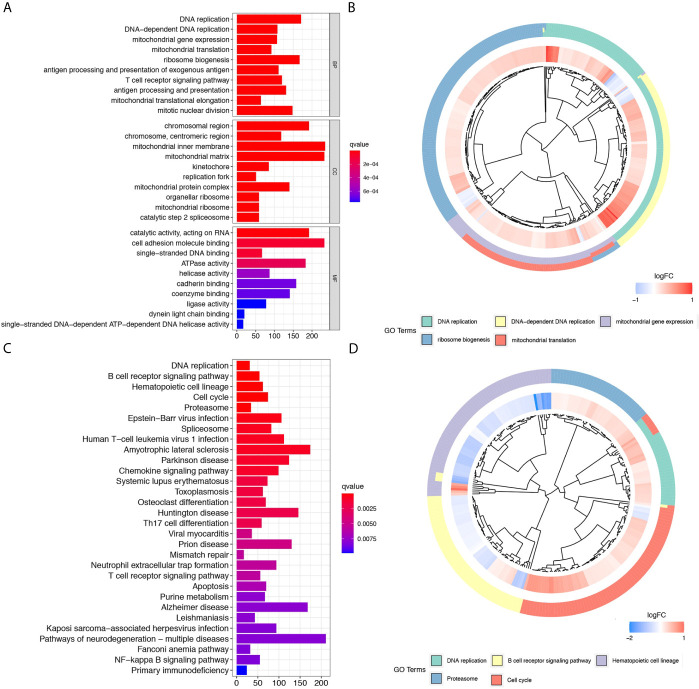
Further comparison between high- and low-risk groups of the TCGA cohort. **(A)** and **(B)** show the bar plot and cluster plot of significant GO functional items, respectively. **(C)** and **(D)** show the bar plot and cluster plot of significant KEGG pathways, respectively.

GO functional enrichment analyses, and KEGG pathway enrichment analyses were performed on the DElncRNAs between the high- and low-risk groups. The GO analysis results indicated that the 12 prognostic lncRNAs mainly focused on DNA replication, DNA-dependent DNA replication, ribosome biogenesis, and mitochondrial gene expression (**Figures 4A, B** **)**. The KEGG analysis results show that the 12 prognostic lncRNAs are mainly enriched in DNA replication pathway, B cell receptor signaling pathways, hematopoietic cell lineage pathway, and cell cycle pathway (**Figures 4C, D** **)**.

### Immunity Analyses

**Figure 5:**
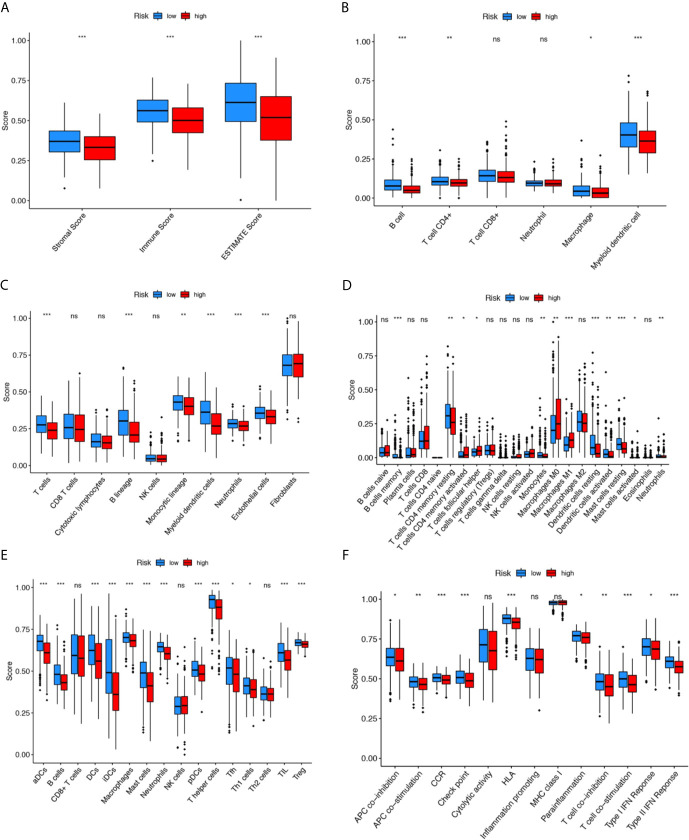
**﻿**Comparison of the immunity analysis between different risk groups. **(A–F)** indicates the ESTIMATE, TIMER, MCP counter, CIBERSORTx, and single-sample gene set enrichment analysis (ssGSEA) algorithms to compare the cellular components or cell immune responses between two groups. ﻿CCR, cytokine-cytokine receptor. Adjusted P values were showed as: ns, not significant; *P < 0.05; **P < 0.01; ***P < 0.001.

To further explore the relationship between the LUAD prognosis and immune status, we quantified the infiltrating scores of immune cell- and immunity-related functions in two groups with ESTIMATE, TIMER, MCP counter, CIBERSORTx, and single-sample gene set enrichment analysis(ssGSEA) algorithms. There was a remarkable difference between the two groups, as shown in **Figure 5**. The ESTIMATE score was significantly higher in the low-risk group than in the high-risk group, indicating a higher overall immune level and immunogenicity of the tumor microenvironment (TME) in the low-risk group (p<0.001). Correlation analysis of immune cell subpopulations based on ssGSEA of TCGA-LUAD data revealed that the scores of immune cell, including aDCs, B cells, DCs, iDCs, Macrophages, NK-cells, T helper cells and Treg were significantly different between the low- and high-risk groups (p<0.001). In addition, there were significant differences in immune function scores including APC co-stimulation, inflammation promoting, check point, T cell co−inhibition T cell co−stimulation and Type II IFN Response between the low-risk and high-risk groups (p<0.01).

## Discussion

LUAD is characterized by a high degree of heterogeneity, and studies have demonstrated that a variety of genetic mutations and epigenetic changes drive the development and progression of LUAD, including gene fusion, major chromosomal events, single nucleotide changes (SNVs) and copy number alterations (CNAs) (36, 37). In recent years, with the in-depth understanding of the changes in the molecular mechanism of LUAD, many molecular targeted drugs for the precise treatment of LUAD have been developed, such as tyrosine kinase inhibitors (TLIs) of epidermal growth factor receptor (EGFR) and PDL1/PDL inhibitors (38, 39), which bring hope to LUAD patients. However, these mutations are not ubiquitous in all LUAD patients, and existing targeted drugs are only effective in some patients, and even some patients develop resistance and relapse during treatment. Therefore, further investigation of possible molecular mechanisms in LUAD is needed to provide new targets and biomarkers for individualized treatment and prognosis of LUAD. Recently, studies have demonstrated that the therapeutic effect of tumors can be improved by regulating the cell death process (40). Ferroptosis is an iron-dependent cell death program that has been shown to be involved in the development of tumors and the response to anti-tumor treatment (14, 19, 41). In addition, many lncRNAs are known to participate in malignant tumor progression and tumor resistance, and become new biomarkers and therapeutic targets for cancer diagnosis and treatment (42–44). Many lncRNAs can affect the ferroptosis of cancer cell and tumor immunity (45–47), being promising to become tumor markers with clinical value.

LncRNAs have been found to play a key role in ferroptosis of cancer cells. For example, lncRNAs engage the development and progression of NSCLC by regulating ferroptosis process (48). SLC7A11, the target gene of lncRNA, is a key gene of ferroptosis that can be downregulated by XAV939 (an NSCLC inhibitor), which inhibits the development of NSCLC through a ferroptosis mediated pathway (49). Jie Wu et al. found that lncRNA APCDC1L-AS can induce LUAD resistance to icotinib (a kind of TLIs) by inhibiting autophagic degradation of EGFR (44). The lncRNA P53RRA in the cytoplasm affects some metabolic gene transcription and promotes ferroptosis by activating P53 (45). P53RRA enhances erastin-induced growth inhibition while also increasing the concentration of intracellular iron and lipid ROS, which correlates with ferroptosis in NSCLC. LncRNALINC00336 is a key suppressor of ferroptosis and exerts antitumor effects in lung cancer by reducing intracellular iron and lipid ROS levels through interaction with ELAVL1 (50). These evidences suggest that ferroptosis-related lncRNAs are key regulators of ferroptosis and may be effective targets and specific markers for LUAD treatment and prognosis.

Here, we explored the association between ferroptosis-related lncRNAs and LUAD prognosis. The LUAD risk prediction model was successfully constructed with 12 ferroptosis-related lncRNAs, including five protective factors (C5orf64, LINC01800, LINC00968, LINC01352, PGM5-AS1) and seven risk molecules (LINC02097, DEPDC1-AS1, WWC2-AS2, SATB2-AS1, LINC00628, LINC01537, LMO7DN).C5orf64, LINC00968, WWC2-AS2, LINC00628, and LMO7DN have been shown to be associated with progression or prognosis of LUAD. We found that C5orf64 was the most significant protective factor, and Zhaofei Pang et al. found that C5orf64 was also an immune-related lncRNA that positively correlated with M2 macrophage, monocyte, eosinophil, and neutrophil infiltration in TIME of LUAD, but negatively correlated with Tregs and plasma cell infiltration; in addition, PD-1, PD-L1, and CTLA-4 were highly expressed in patients with high levels of C5orf64 (51). LINC00968 was prove to be associated with tumor progression and drug resistance (52–55). LINC00968 is under expressed in LUAD and inhibits tumor cell growth through the linc00968/miR-9-5p/CPEB3 (56) and miR-21-5p/SMAD7 (57) axes.LINC00628 is a tumor suppressor gene in multiple tumors, including gastric cancer (58), breast cancer (59), osteosarcoma (60) and hepatocellular carcinoma(HCC) (61). However, it was found that LINC00628 silencing inhibited proliferation, migration, and invasion of LUAD cells (62),which is consistent with our findings that LINC00628 acts as a risk factor for LUAD. Yonathan Brhane et al. identified that LMO7DN was associated with LUAD prognosis at the genome-wide level (63), suggesting that LMO7DN may play an important role in LUAD progression. Xiuqing Shen et al. demonstrated that WWC2-AS2 can be used as a predictor of LUAD, but its role in LUAD is unknown (64). Although there is little evidence of direct association of LINC01352, PGM5-AS1, and SATB2-AS1 with LUAD, many studies have shown that they play a key role in different tumors. Pinbo Huang et al. found that HBV/HBx (HBV X protein) complex can reduce the expression level of LINC01352 which in turn inhibits LINC01352-miR-135b-APC axis and promotes the development of HCC.LINC01352 is an independent prognostic molecule for HCC (65). PGM5-AS1 has different effects in different tumors. For example, PGM5-AS1 upregulates GDF10 gene expression by competitively binding to miR-587, inhibiting prostate cancer cell growth and accelerating apoptosis (66). In contrast, PGM5-AS1 can weaken the inhibitory effect of fibrillin-1 which mediated by miR-140-5P, and promote epithelial-mesenchymal transition, invasion and metastasis of osteosarcoma cells (67). Low SATB2-AS1 expression is associated with poor prognosis in colorectal cancer (CRC), and it can inhibit CRC metastasis and regulate TH1-type chemokine expression in TIME (68). However, we have not yet found studies on the significance of LINC02097, DEPDC1-AS1, and LINC01537 in LUAD or other tumors. Our findings demonstrate for the first time that these three lncRNAs are associated with the prognosis of LUAD, and their mechanism of action in LUAD needs to be explored.

In addition, we constructed the LUAD risk model with good predictive power and robustness, and it can be used as an independent prognostic factor for LUAD. This predictive model can effectively classify patients into low-risk group and high-risk group. Whether in the training cohort or validation cohort, we found a better prognosis in the low-risk group, which illustrates that 12 ferroptosis-related lncRNAs may be good prognostic factors for LUAD.

Due to different genetic mutations in different patients, targeted therapy is more likely to be a precise individualized treatment. We need to understand the molecular mechanisms that may occur in LUAD and find new therapeutic targets. Therefore, we further revealed the possible signaling pathways involved in ferroptosis-related lncRNAs by functional enrichment analysis, which are associated with a variety of life activities of cells, including cellular immunity, cell cycle, DNA replication, mitochondrial function and so on, indicating that ferroptosis-related lncRNAs may be involved in the development and progression of LUAD. The effect of immunotherapy depends on the immunogenicity of TME, so understanding TIME is the key to evaluating immunotherapy (69). By immune infiltration analysis, comparing to high-risk group, we found that the abundance of immune cells (dendritic cells, B cells, CD8 + cells, macrophages and neutrophils) in low-risk group was higher. Besides, a variety of immune system process pathways are involved, suggesting that low-risk patients have higher immunogenicity in TEM and better response to immunotherapy. The results of our study may help guide immunotherapy for LUAD.

However, our study has some shortcomings. First, based on traditional statistical methods, we constructed and evaluated 12 ferroptosis-related lncRNA risk prediction models. Although many studies have demonstrated the feasibility of these methods, more high-level techniques and methods need to be developed to improve the accuracy and robustness of prognostic models. In addition, this model has only been validated in GEO datasets, and external and practical validation is needed in the future to evaluate whether it can be applicable to clinical patients. Finally, we only preliminarily explored the signaling pathways involved in ferroptosis-related lncRNAs, however, the specific mechanism of ferroptosis-related lncRNAs in LUAD and their interconnection with TIME and ferroptosis are not yet fully understood, and more studies are needed to validate our findings.

## Conclusion

In conclusion, we found 12 ferroptosis-related lncRNAs associated with LUAD prognosis. A risk score constructed based on these 12 biomarkers, can independently predict the prognosis of LUAD patients. Furthermore, by functional enrichment analysis, we explored the role of these 12 biomarkers in the immunity and ferroptosis of LUAD, providing new insights for further understanding of the molecular mechanisms in the development and progression of LUAD. These biomarkers have many potential therapeutic and prognostic implications for LUAD patient management.

## Data Availability Statement

Publicly available datasets were analyzed in this study. This data can be found here: The Cancer Genome Atlas database (https://portal.gdc.cancer.gov/) and Gene Expression Omnibus database (https://www.ncbi.nlm.nih.gov/geo/).

## Author Contributions

RG and Q-YZ conceived and designed the study. LL and L-PL provided equal contributions to research design, data analysis and article writing. Q-QZ revised the manuscript. All authors contributed to the article and approved the submitted version.

## Funding

This study was supported by the National Natural Science Foundation of China (Grant No. 81573091) and the Fundamental Research Funds for the Central Universities of Central South University under Grant (No. 2020zzts892; 2021zzts1093).

## Conflict of Interest

The authors declare that the research was conducted in the absence of any commercial or financial relationships that could be construed as a potential conflict of interest.
