# Supplementary material for: Identification of a Ferroptosis-Related LncRNA Signature as a Novel Prognosis Model for Lung Adenocarcinoma
Source: Front Oncol. 2021 Jun 23;11:675545. doi: 10.3389/fonc.2021.675545 (PMC8260838; doi:10.3389/fonc.2021.675545)
Supplement: Supplementary file 2 [file Table_2.docx]

# Supplementary Figures and Tables

**Figure S1.** Expression levels of ferroptosis-related lncRNA between LUAD and normal samples. Blue through red color indicates low to high expression level.

**Figure S2.** The co-expression relationships between ferroptosis-related lncRNAs (blue points) with their related mRNAs (red points).

**Table S2.** The coefficient of each lncRNA in the risk score

| Coefficient | lncRNA |
| --- | --- |
| -1.99101 | C5orf64 |
| -0.46779 | LINC01800 |
| 0.43414 | LINC02097 |
| 0.42931 | DEPDC1-AS1 |
| 0.33380 | WWC2-AS2 |
| 0.31422 | SATB2-AS1 |
| 0.20384 | LINC00628 |
| 0.19138 | LINC01537 |
| -0.10041 | LMO7DN |
| -0.07110 | LINC00968 |
| -0.07096 | LINC01352 |
| -0.06665 | PGM5-AS1 |

The formula of the risk score was:

RiskScore = coef_1_×ICAG_1_ + coef_2_× ICAG_2_ + coef_3_× ICAG_3_ + ⋯
